# Rearrangement processes and structural variations show evidence of selection in oesophageal adenocarcinomas

**DOI:** 10.1038/s42003-022-03238-7

**Published:** 2022-04-08

**Authors:** Alvin Wei Tian Ng, Gianmarco Contino, Sarah Killcoyne, Ginny Devonshire, Ray Hsu, Sujath Abbas, Jing Su, Aisling M. Redmond, Jamie M. J. Weaver, Matthew D. Eldridge, Simon Tavaré, Oesophageal Cancer Clinical and Molecular Stratification (OCCAMS) Consortium, Nicola Grehan, Barbara Nutzinger, Elwira Fidziukiewicz, Adam Freeman, Elizabeth C. Smyth, Maria O’Donovan, Ahmad Miremadi, Shalini Malhotra, Monika Tripathi, Calvin Cheah, Hannah Coles, Connor Flint, Matthew Eldridge, Maria Secrier, Sriganesh Jammula, Jim Davies, Charles Crichton, Nick Carroll, Richard H. Hardwick, Peter Safranek, Andrew Hindmarsh, Vijayendran Sujendran, Stephen J. Hayes, Yeng Ang, Andrew Sharrocks, Shaun R. Preston, Izhar Bagwan, Vicki Save, Richard J. E. Skipworth, Ted R. Hupp, J. Robert O’Neill, Olga Tucker, Andrew Beggs, Philippe Taniere, Sonia Puig, Timothy J. Underwood, Robert C. Walker, Ben L. Grace, Jesper Lagergren, James Gossage, Andrew Davies, Fuju Chang, Ula Mahadeva, Vicky Goh, Francesca D. Ciccarelli, Grant Sanders, Richard Berrisford, David Chan, Ed Cheong, Bhaskar Kumar, L. Sreedharan, Simon L. Parsons, Irshad Soomro, Philip Kaye, John Saunders, Laurence Lovat, Rehan Haidry, Michael Scott, Sharmila Sothi, Suzy Lishman, George B. Hanna, Christopher J. Peters, Krishna Moorthy, Anna Grabowska, Richard Turkington, Damian McManus, Helen Coleman, Russell D. Petty, Freddie Bartlett, Paul A. W. Edwards, Rebecca C. Fitzgerald

**Affiliations:** 1 Medical Research Council Cancer Unit, Hutchison/Medical Research Council Research Centre, University of Cambridge, Cambridge, UK; 2 Cancer Research UK Cambridge Institute, University of Cambridge, Cambridge, UK; 3 Institute of Cancer and Genomic Sciences, College of Medical & Dental Sciences, University of Birmingham, Birmingham, UK; 4 University Hospitals Birmingham NHS Foundation Trust, Birmingham, B15 2GW UK; 5 European Molecular Biology Laboratory, European Bioinformatics Institute EMBL-EBI, Hinxton, UK; 6 Department of Surgery, University of Cambridge, Cambridge, UK; 7 Department of Medical Oncology, The Christie NHS Foundation Trust, Manchester, UK; 8 Department of Pathology, University of Cambridge, Cambridge, UK; 9 Irving Institute for Cancer Dynamics, Columbia University, New York, USA; 10 Department of Statistics, Columbia University, New York, USA; 11 Department of Biological Sciences, Columbia University, New York, USA; 12 Cambridge University Hospitals NHS Foundation Trust, Cambridge, CB2 0QQ UK; 13 Department of Histopathology, Addenbrooke’s Hospital, Cambridge, UK; 14 Department of Computer Science, University of Oxford, Oxford, OX1 3QD UK; 15 Salford Royal NHS Foundation Trust, Salford, M6 8HD UK; 16 Faculty of Medical and Human Sciences, University of Manchester, Manchester, M13 9PL UK; 17 Wigan and Leigh NHS Foundation Trust, Wigan, Manchester, WN1 2NN UK; 18 GI Science Centre, University of Manchester, Manchester, M13 9PL UK; 19 Royal Surrey County Hospital NHS Foundation Trust, Guildford, GU2 7XX UK; 20 Edinburgh Royal Infirmary, Edinburgh, EH16 4SA UK; 21 Edinburgh University, Edinburgh, EH8 9YL UK; 22 King’s College London, London, WC2R 2LS UK; 23 University Hospital Southampton NHS Foundation Trust, Southampton, SO16 6YD UK; 24 Cancer Sciences Division, University of Southampton, Southampton, SO17 1BJ UK; 25 Guy’s and St Thomas’s NHS Foundation Trust, London, SE1 7EH UK; 26 Karolinska Institute, Stockholm, SE-171 77 Sweden; 27 Plymouth Hospitals NHS Trust, Plymouth, PL6 8DH UK; 28 Norfolk and Norwich University Hospital NHS Foundation Trust, Norwich, NR4 7UY UK; 29 Nottingham University Hospitals NHS Trust, Nottingham, NG7 2UH UK; 30 University College London, London, WC1E 6BT UK; 31 Wythenshawe Hospital, Manchester, M23 9LT UK; 32 University Hospitals Coventry and Warwickshire NHS Trust, Coventry, CV2 2DX UK; 33 Department of Surgery and Cancer, Imperial College, London, W2 1NY UK; 34 Queen’s Medical Centre, University of Nottingham, Nottingham, UK; 35 Centre for Cancer Research and Cell Biology, Queen’s University Belfast, Belfast, BT7 1NN Northern Ireland; 36 Tayside Cancer Centre, Ninewells Hospital and Medical School, Dundee, DD1 9SY Scotland; 37 Portsmouth Hospitals NHS Trust, Portsmouth, PO6 3LY England

**Keywords:** Oesophageal cancer, Cancer genomics, Oncogenes, Tumour-suppressor proteins

## Abstract

Oesophageal adenocarcinoma (OAC) provides an ideal case study to characterize large-scale rearrangements. Using whole genome short-read sequencing of 383 cases, for which 214 had matched whole transcriptomes, we observed structural variations (SV) with a predominance of deletions, tandem duplications and inter-chromosome junctions that could be identified as LINE-1 mobile element (ME) insertions. Complex clusters of rearrangements resembling breakage-fusion-bridge cycles or extrachromosomal circular DNA accounted for 22% of complex SVs affecting known oncogenes. Counting SV events affecting known driver genes substantially increased the recurrence rates of these drivers. After excluding fragile sites, we identified 51 candidate new drivers in genomic regions disrupted by SVs, including *ETV5, KAT6B* and *CLTC. RUNX1* was the most recurrently altered gene (24%), with many deletions inactivating the RUNT domain but preserved the reading frame, suggesting an altered protein product. These findings underscore the importance of identification of SV events in OAC with implications for targeted therapies.

## Abstract (web summary)

Genomic structural variants (SVs) in cancer are notoriously difficult to identify, despite the impact they have on cancer progression and treatment efficacy. This study applies state-of-the-art computational methods to catalogue SVs in 383 cases of oesophageal cancer, uncovering mutational patterns and new disease drivers.

## Introduction

Patterns of rearrangement can reflect the underlying mechanism generating the rearrangement, genetic instabilities or mutagen exposures, and these may in turn determine response to therapy or help explain the underlying aetiology^1,2^. Rearrangements in driver genes, such as deletions, amplifications, gene breakages and gene fusions, seem likely to be at least as important a source of driver mutations as single nucleotide variants (SNVs) and indels in many carcinomas^3–5^. The Pan-Cancer Analysis of Whole Genomes (PCAWG) analysed whole genome sequencing data from multiple cancer types and this revealed a remarkable heterogeneity of SVs. In some cancer types, such as breast and ovary, it was estimated that up to three times more driver genes are altered by SVs than by SNVs and indels^3^. Nevertheless, our ability to identify SV driver events lags far behind that of SNV and indel events. This is primarily because there is no measure of the background SV mutation rate, unlike synonymous SNV mutations, that enable the identification of driver genes disrupted by SNVs and rearrangements often involve large genomic regions^6,7^.

Oesophageal cancer, especially the subtype oesophageal adenocarcinoma (OAC), emerged from the PCAWG analysis (*n* = 100 OACs) as a cancer type with one of the highest burdens of SVs with complex rearrangements^1,3^. These include breakage-fusion-bridge (BFB) cycles; catastrophic chromothripsis events with oscillating copy number patterns^8^, deletions in the fragile-sites and the highest rate of somatic mobile element (ME) inserts of any cancer type^1,9–12^. MEs are mainly inserts from Long Interspersed Nuclear Element-1 (LINE-1) retrotransposons, and can consist either of LINE-1 sequence alone or LINE-1 with up to a few kb of 3′ flanking unique genomic sequence transduced^11,13^.

Driver alterations in SNVs and indels are well characterized in OAC, as are distinct copy number (CN) amplification of oncogenes (e.g. *ERBB2, EGFR, RB1, GATA4/6, CCND1* and *MDM2*) and loss of tumour suppressors (e.g. *TP53, CDKN2A, CDKN2B*)^9,10,14^. Rearrangement processes such as BFB cycles and extrachromosomal circular DNA (ecDNA) have been shown to result in copy number amplification in key oncogenes^15–18^ while a variety of SVs can disrupt tumour suppressor genes, including LINE-1 insertions^11,14^. However, to date the analysis of these complex events in OAC has not been performed at the detail required to fully elucidate the spectrum and underlying mechanisms for complex SVs.

In this analysis we combine recent advances in methods for dissecting complex rearrangements and identifying driver events^1,3,11,19–22^ to characterize SVs in a large cohort of 383 OACs with paired whole transcriptome sequence (WTS) in a subset (*n* = 214). Coupled with detailed clinical annotation, this analysis has enabled us to establish the functional relevance of the driver genes affected by these rearrangements.

## Results

### Rearrangement patterns in OAC genomes show frequent mobile element insertions and complex SV

**Fig. 1:**
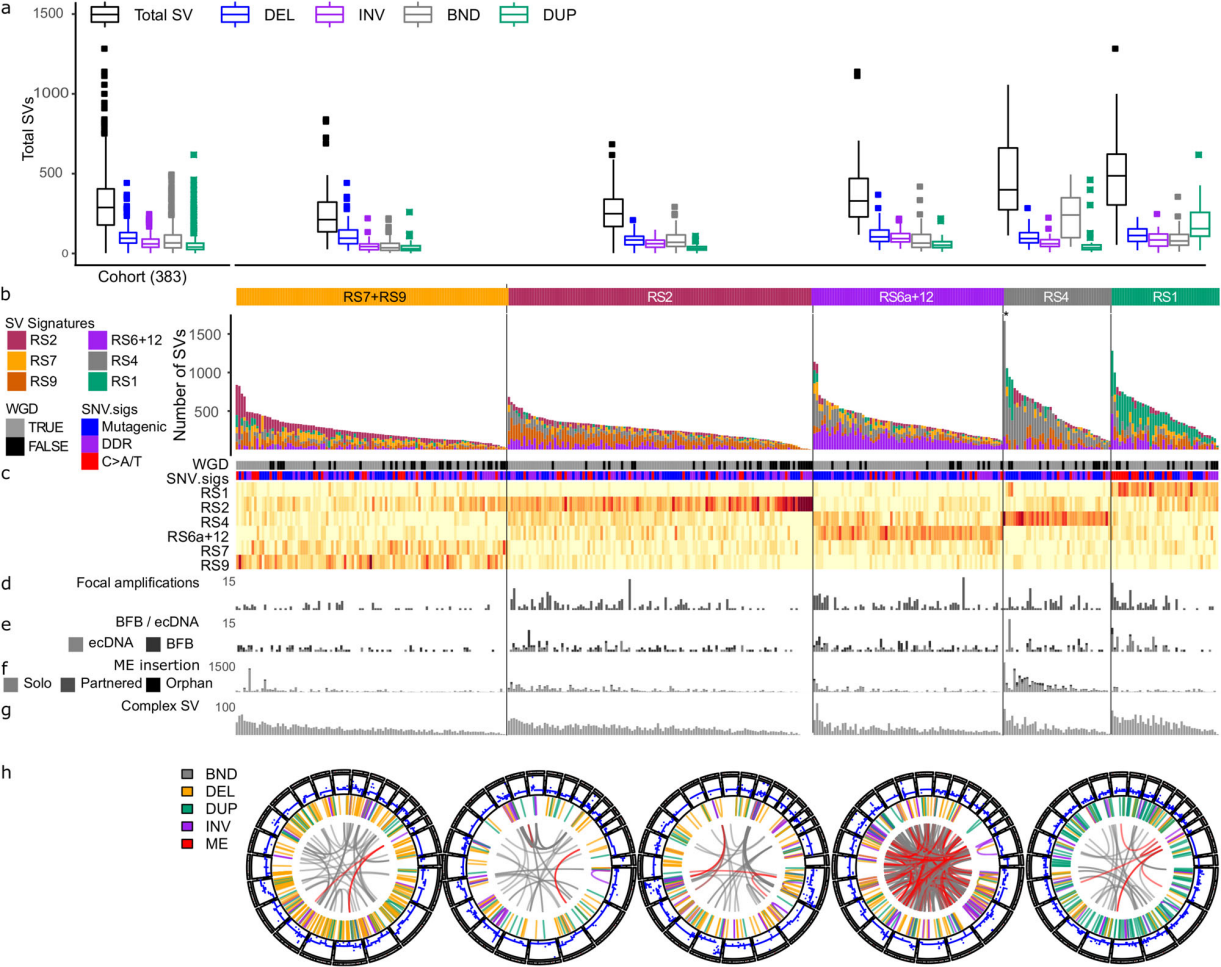
Classification of OACs according to the proportions of SV types and signatures. Tumours are shown classified into groups according to their predominant SV signature defined by Nik-Zainal et al. (2016). **a** Box plot showing numbers of SVs by SV type for the entire cohort and in each group (named after the simplest rearrangement that could generate such a junction, DEL: deletion, INV: inversion, BND: ‘breakend’, i.e. an inter-chromosome junction or translocation, DUP: tandem duplication). **b** Bar plots of rearrangements associated to each rearrangement signatures in OAC. **c** Heatmap showing proportions of SVs associated to each signature and a comparison with related variables: whole genome doubling (WGD), SNV signature classification (Mutagenic, DDR and C > A/T) described by Secrier (2016), **d** focal amplifications, **e** number of BFB and ecDNA cycles, **f** number of mobile element insertions and **g** complex SV clusters. **h** Circos plots of representative tumours from each signature group with ME insertions highlighted in red. *Denotes tumour with >2500 SVs excluded from plot.

We analysed 383 OAC genomes and observed a wide variation in the numbers of structural variants (SV) between cases, with a predominance of deletions (DEL), inter-chromosome junctions (BND) and tandem duplications (DUP) (Fig. 1a). The SV were deconvoluted into rearrangement signatures (RS, Supplementary Fig. S1A) by combining the types of SVs with the size and degree of clustering^2,23^, mapped to known signatures (Supplementary Fig. S1B)^24^ and clustered to show distinct profiles of rearrangements in different groups of patients (Fig. 1b, c, Supplementary Fig. S2A, Supplementary Data 1). Six RS were identified: two with DEL sizes of 1–10 kb and 100 kb–1 Mb (signatures RS7 and RS9, respectively); a non-clustered inter-chromosomal junction (BND) (RS2) and a clustered inter-chromosomal junction signature (RS4); and a clustered SV signature with a high number of DELs, INVs, and DUPs of size 1–10 Mb, corresponding to a combination of signatures (RS6a and RS12) and a non-clustered 100 kb–1 Mb DUP signature RS1^24^. We identified a lower burden of focal amplifications and extrachromosomal DNA (ecDNA) cycles in the RS7 + RS9 group (*p* = 0.0056, *p* = 0.0061, respectively, Wilcoxon rank sum test, Supplementary Fig S2B, C), an enrichment of mobile element (ME) insertions in the RS4 group (*p* = 4 × 10^−11^) and complex clusters of SVs in the RS1 group of patients (*p* = 8.2 × 10^−7^, Fig. 1d–g, Supplementary Fig. S2B–G).

To determine the contributions of ME insertions in generating SVs in OAC, we used the TraFic algorithm^11,13^, which identified a median of 60 (IQR 3–117) ME inserts per tumour (Fig. 1f, Supplementary Data 2). The majority of inserts (81%, 37,475) were of LINE-1 sequence alone (‘solo’), while 19% (8517) included transduced 3′ flanking sequence. Of these 7% (3195) retained LINE1 sequence, while 12% (5322) were ‘orphan’ transductions, i.e. transduced sequence alone (Fig. 1f, Supplementary Data 2). Since transduced sequence reveals the origin of the LINE-1 in the genome, we could assign 13% (6109) to germline elements and, remarkably, 5% (2408) to novel, somatically acquired elements. In the tumours with the highest numbers of inserts, the active germline LINE-1s were generally those described by Rodriguez-Martin et al. as ‘Plinian’, i.e., rarely present but with high activity when activated. This is in contrast to the ‘Strombolian’ germline LINE-1 elements, which are frequently active in cancer and tend to be active in tumours with fewer inserts^3,11^. We also identified ME insertions among our conventional SV calls and as most are inter-chromosomal, most resemble translocations. Hence, there were 13,189 inter-chromosomal junctions that had at least one breakpoint overlapping with a ME called by TraFic in the sample (Supplementary Data 2).

### Rearrangement signatures in OAC correspond to processes leading to ME insertions, DNA damage repair and complex rearrangements

To identify the features of biological processes associated with each RS, we carried out a logistic regression based on the presence of each RS in each tumour and orthogonal features including the number of ME insertions; chromothripsis events, complex SV clusters, SNV signatures subtypes^10^, BFB or ecDNA events numbers and in known driver genes (Supplementary Data 3).

RS4, a signature of unknown aetiology consisting of clustered inter-chromosomal junctions (affecting 74% of cases), was strongly associated with the number of ME insertion events (log odds: 6.13, *p* = 3.21 × 10^−9^, Supplementary Data 4). We further determined if each inter-chromosomal junction cluster overlapped with nearby ME insertions or source elements and found 59% (1622/2751) of RS4 clusters overlapped with ME insertions—41% called by TraFic, while the remaining 18% of RS4 clusters overlapped with regions with previous evidence of transductions by MEs^11,13^ (Supplementary Data 5). We also identified an association with the number of ecDNA amplicons (log-odds = 0.46, *p* = 0.009, Supplementary Data 4) and increased KRAS expression (log odds 0.54, *p* = 5.47 × 10^−4^, logistic regression, *p* = 0.026, Wilcoxon rank sum test) in tumours with RS4, driven by tumours (15/19) with both ME insertions and *KRAS* amplification (Supplementary Fig. S2H, Supplementary Data 6). In addition, RS4 was associated with a lower expression of Leucine Rich Repeat Kinase 2 (*LRRK2*), a gene with interactions with *ATM* and roles regulating *MDM2* and *TP53* in DNA repair pathways^25^ that was previously identified^14^ (log odds = −0.99, *p* = 4.91 × 10^−4^, Supplementary Data 4). In addition, we observed that tumours with RS4 had an increased frequency of SVs in genomic regions containing *MDM2, H3F3B, PTPRB* and *GRM3* compared to tumours devoid of RS4 (Supplementary Fig. S2I).

Signature RS2 (87%) was associated with a lower number of ecDNA amplicons involving *ERBB2* (log odds −0.89, *p* = 0.033, FDR = 0.051, Supplementary Data 4). Tumours with a high proportion of SVs assigned to RS2 have a low burden of SV events (*p* = 0.0181, Wilcoxon rank sum test, Supplementary Fig. S2A) and are genomically stable compared to other tumours.

The deletion signature, RS7 (69%) was associated with an absence of ME insertions (log odds = −1.53, *p* = 2.85 × 10^−8^) and a higher burden of the SNV signature SBS17a (log odds = 0.55, *p* = 3.33 × 10^-6^). RS9 (68%) was associated the presence of the DNA damage response (DDR) phenotype based on SNV signatures described by Secrier et al. ^10^ (log odds 1.41, *p* = 1.71 × 10^−4^) and a lower number of ecDNA cycles affecting the cell cycle regulator Cyclin E1 (*CCNE1*, log odds = −1.14, *p* = 0.025, Supplementary Data 4).

Signature RS1 (47%) was associated with ecDNA events encompassing (log odds 3.28, *p*-value = 0.004, logistic regression, Supplementary Data 4) and increased expression of *CCNE1* compared to other tumours (log odds 0.88, *p*-value = 1.55 × 10^−4^, logistic regression, Supplementary Data 4, *p* = 7.5 × 10^-7^, Wilcoxon rank sum test, Supplementary Fig. S2J). Tumours with RS1 were associated with an absence of ecDNA spanning *CDK6* (log odds −1.18 *p* = 0.008) and low ME insertions (−1.14, *p* = 2.55 × 10^-4^, Supplementary Data 4). RS1 corresponded to the tandem duplication phenotype signature, associated with high *CCNE1* expression (*p* = 3.6 × 10^−6^, Wilcoxon rank sum test, Supplementary Fig. S2J) and replication stress, previously reported in breast, ovarian, stomach and liver cancer^2,26–28^

The ‘clustered’ signature RS6a + RS12 (69%) was associated with complex SV including a higher number of ecDNA and BFB cycles (log odds = 0.47, *p* = 0.003, log odds = 0.69, *p* = 9.19 × 10^−5^, respectively). Complex rearrangements consisting of clustered inversions and foldback inversions made up 20% of SV clusters associated with RS6a + RS12, and many additional clusters containing larger complex events (Fig. 1e, g).

Five example tumours are shown, respectively, with a high proportion of predominant deletions (RS7 + RS9); non-clustered SVs (RS2); densely clustered SV inversions (RS6 + 12); inter-chromosomal junctions overlapping LINE-1 ME insertions (RS4) and clusters of tandem duplications (RS1) (Fig. 1h).

### Complex SVs involving known oncogenes in OAC can be explained by ecDNA amplicons

Complex clusters of rearrangements are thus a prominent feature in OAC and we sought to identify clusters which are likely due to the formation of BFB cycles consisting of foldback inversions and circular ecDNA events that alter known oncogenes. We identified ecDNA events by applying the Amplicon Architect tool^15^, that starts from regions estimated by CNVKit^29^ to have an absolute copy number > 4.5 and segment size > 50 kbp and searches for additional regions in the genome that are joined to form an amplicon. As ecDNA events can arise from BFB events and Amplicon Architect identifies BFB, BFB-linked cyclic amplicons and cyclic amplicons, we grouped these events as BFB or ecDNA amplicons. We identified 507 BFB or ecDNA amplicons, of which 58.2% (295) encompassed oncogenes known to be drivers in OAC, accounting for 22% of complex SV clusters overlapping a known oncogene (Supplementary Data 5). Among these ecDNA or BFB events, at least 13 showed inclusion of regions with H3K27Ac marks (*p* = 0.0002, regioneR^30^, permutation test) identified in OAC cell lines and tumours^31^ and devoid of genes—most notably, 4 enhancer elements on chromosome 17 amplified and part of amplicons involving *CDK12, ERBB2, RNF43 and CLTC* (Supplementary Data 7, Supplementary Fig. S3).

**Fig. 2:**
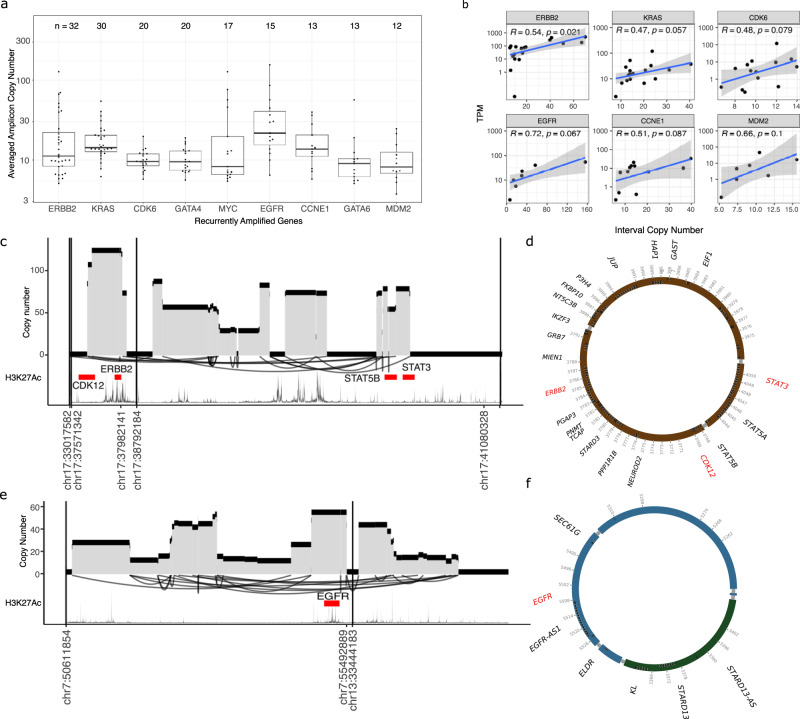
Complex SVs leading to amplification of oncogenes. **a** Recurrent amplicons detected by Amplicon Architect associated with known OAC oncogenes. The number of tumours with detected amplicon is shown above. *Y*-axis showing copy number of segments spanning each gene, averaged along the length of segment. **b** Correlation of gene expression (TPM) and copy number of amplicons. **c** Example of an amplified region spanning CDK12, ERBB2, STAT3 and STAT5B, resembling ecDNA and **d** Reconstructed amplicon as an extrachromosomal circle containing ERBB2 and a CDK12-STAT5B fusion. **e** An amplified region spanning EGFR and joining chromosomes 7 and 13, forming an ecDNA and reconstructed as a circle (**f**).

Known driver genes in OAC were recurrently amplified through BFB or ecDNA amplicons, with 35.5% (136) patients having one or more amplicons encompassing *ERBB2, KRAS, CDK6, GATA4, MYC, EGFR, CCNE1, GATA6* or *MDM2* (Fig. 2a, Supplementary Data 8). Amplicons showed a wide variation of copy number (median CN = 12, IQR 7.9–19.1) and positive correlation (Pearson’s correlation = 0.42, *p* = 2.993 × 10^−5^) with high gene expression (Fig. 2b). In addition, several likely driver genes were co-amplified in large complex amplicons, notably *CCR7*, and/or *RARA*co-amplified with *ERBB2* (Fig. 2c, Supplementary Fig. S3); and *AKAP9* and/or *GATAD1* with *CDK6* (Supplementary Fig. S3).

It is instructive to consider individual cases. For example, in a tumour with 28 SV breakpoints in two clusters around the highly amplified *CDK12-ERBB2* (copy number = 115) and *STAT5B-STAT3* (copy number = 72) loci, reconstruction suggested there were multiple ecDNA circles or segments carrying either *ERBB2* or *STAT5B* alone, plus some carrying both amplicons (copy number = 25). The combined structure was consistent with a circular ecDNA structure (Fig. 2c, d) that included two clusters of enhancers (hg19/chr17:37773759-37939651, chr17:39768677-39852129). The enhancers were identified in publicly available OAC tumours and OAC cell lines data^31^ and the ecDNA encoded a *CDK12-STAT5B* fusion, that was confirmed using RNA sequencing. Similarly, an *EGFR-SEC61G* fusion previously predicted from DNA sequencing in a PCAWG study^22^, proved to be in an EGFR amplification that was part of a cyclical ecDNA with enhancer marks on both chromosome segments (chr13:33846776-33860433, chr7:55132499-55154521, Fig. 2e, f).

### Identifying SVs in OAC driver events

**Fig. 3:**
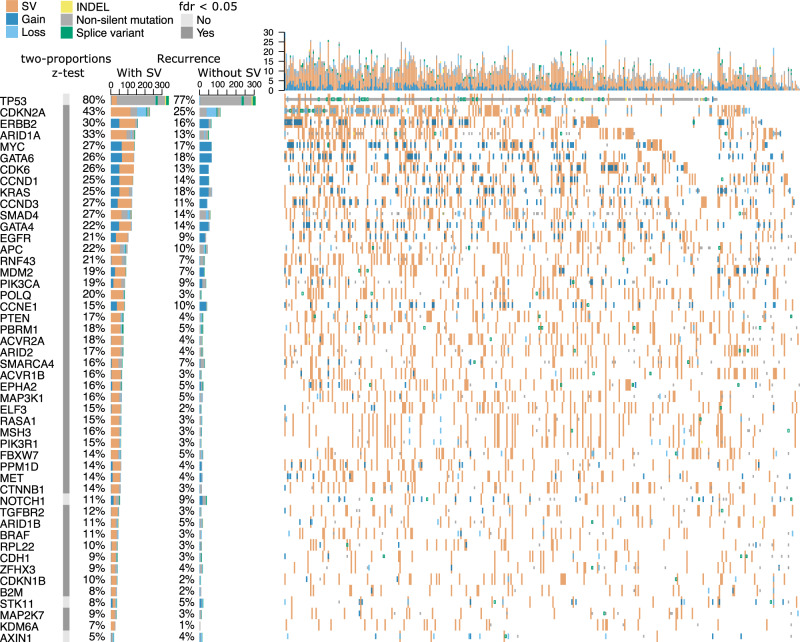
Estimates of recurrence in known driver alterations with and without SVs. Oncoplot showing recurrence of known OAC driver gene mutations (taken from Frankell et al., 2019 and Campbell et al., 2020) with and without SV. Estimates of recurrence without SVs includes copy number gains and losses, INDELs and SNVs. Recurrence with SVs are counted when the interval between two breakpoints overlaps with exon or exons of the gene. Two-proportions *z*-test with multiple hypothesis testing (FDR) used to test if recurrence is significantly higher with SVs included.

To assess the contribution of SVs to driver events we first considered genes that we had previously identified to be targets of SNV, indel, amplification and deletion driver events^3,14^. We identified likely additional driver events due to SVs where the interval between two breakpoints overlapped an exon or exons of known driver gene. Adding these SV events substantially increased the recurrence rates of known drivers. For example, among major tumour suppressors, recurrence *CDKN2A* increased from 25% to 43% and *SMAD4* from 14% to 27%, *PTEN* from 4% to 17% and *APC* to from 10% to 22% while TP53 showed a predominance of SNV alterations (Fig. 3, Supplementary Data 9).

We carried out a two-proportions*z*-test to compare the recurrence of all 48 canonical drivers (*p*-value = 2.2 × 10^−16^) and in each individual gene, with and without considering SVs. Aside from four genes (*TP53, AXIN1, NOTCH1, STK11*) known to be affected by SNVs, 44 out of 48 genes show a significantly higher recurrence when SVs are considered (Supplementary Data 9).

**Fig. 4:**
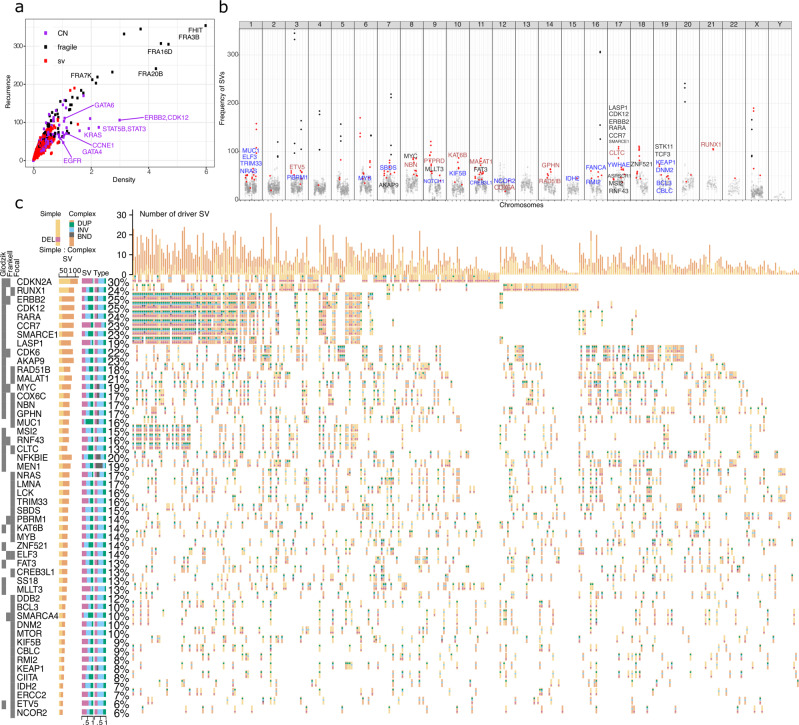
Recurrence and density of SVs in 1 Mb genomic bins. **a** Scatter plot showing recurrence, the number of patients with an SV break in each 1 Mb bin (*y*-axis) and density, the average number of SV breaks in the bin over all tumours (*x*-axis). Bins are labelled with genes or fragile sites that they overlap: black, fragile sites; purple, intervals of amplification and deletion; red, putative genes under selection. **b** Manhattan plot showing 1MB bins containing putative drivers (red) and fragile sites (black) and genes coloured by methods discovered: Glodzik model adjusting for genomic context (Black), Focal (F, blue) and both methods (brown: FG). **c** Oncoplot showing candidate driver genes identified using focal and Glodzik methods and annotated if each gene was found in Frankell et al. (2019). Horizontal bar plots show total number of simple (light orange) and complex (dark orange) SVs found in the given gene; proportions of SVs classified as simple that are of the various SV types; and similarly for SVs classified as complex. Each oncoplot cell shows if each patient has a simple or complex SV and the combination of SV types.

Next, we attempted to identify OAC driver genes affected by SVs, or “hotspots”, characterized by more frequent breaks per unit of genome (1 Mb bins, 500 kb overlapping), after removing known fragile sites, and regions flanking amplicons and deletions. By comparing the recurrence and density of SVs in each hotspot, we identified that fragile sites and copy number altered hotspots obscured driver genes affected by SVs and selected a method that adjusts for CN alterations and other genomic context (Fig. 4a). We identified hotspots in two steps, the first using a previously published method that accounts for genomic context^32^. Secondly to find focal SVs, we used a consensus approach where bins had to be identified in at least two of the following methods: (1) background distribution modelling of SVs in a whole-genome, (2) per-chromosome context and (3) rank-sum*k*-means clustering (see the “Methods” section). We further required that the genes to be listed as cancer-relevant by the CGC/COSMIC database.

A total of 108 regions (1 Mb bins, or groups of adjacent bins) with frequent breaks were identified in either the genomic-context dependent model or focal approach and 41 regions contained known COSMIC genes (Fig. 4b, Supplementary Data 10). These included bins containing *RUNX1, MALAT1, RAD51B, COX6C, GPHN, NBN, KAT6B, CLTC, ETV5* and *PTPRD* that were identified by both approaches (Fig. 4b, c, Supplementary Data 10, 11). We noted that *PTPRD* and *GPHN* were identified as genes in possible fragile sites^33,34^ and excluded them from further analyses. As the COSMIC genes present in hotspots might not be directly affected by SVs, we narrowed down driver gene candidates using the criteria of the SV spanning or overlapping the gene by intersecting the genomic region between the start and end position for intra-chromosomal SVs and between the start and end of both breakpoints in an inter-chromosomal breakend (defined by MANTA) with exons of a gene. Sixty-one candidate genes were identified with *RUNX1* as the most recurrent deleted as many of the SVs in the regions overlapped the gene (Fig. 4b, c), and this is discussed in detail below.

Aside from *RUNX1, CDKN2A, BCL3* and *MYB* were identified, with predominant focal deletions affecting *CDKN2A* and duplications affecting *BCL3* and *MYB* (Supplementary Fig. S4). The *MYB* proto-oncogene, originally found as the retroviral oncogene myeoloblastosis B, is a driver not previously identified in OACs through SNV and CN analyses. Duplications overlapping *MYB* span the gene and the evidence of ecDNA events in four patients (CN = 5–45) support its role as an oncogene as identified in other cancer types.

Of the candidate genes identified, 10 were already known as OAC drivers, leaving 51 candidate SV OAC drivers (Fig. 4c, Supplementary Data 11). We classified each rearrangement using ClusterSV^1^ as simple (a single rearrangement not belonging to a cluster) or complex (multiple rearrangements forming a cluster) and the type of alteration. To accurately estimate the prevalence of rearrangement overlapping with each gene, we identified intra-chromosomal regions spanning each pair of breakpoints and the genes lying within each region. This was done to capture oncogenes which are generally comprised within breakpoints in SVs that lead to amplification (i.e, DUPs, INVs or BNDs) or deletion (mainly DELs). A clear pattern emerged where simple alterations affected tumour suppressors genes including *CDKN2A, ARID1A, SMAD4* and *RUNX1*, while complex clusters tended to affect oncogenes (*ERBB2, CDK6, GATA4, GATA6*) often involving amplifications (Fig. 4c). In addition, breaks within known tumour suppressor genes *CDK12*, *ZNF21* and *RNF43* were observed (Figs. 2c, 4c) and have been shown to result in loss in function (Supplementary Fig S4).

We curated genomic regions identified in our SV driver analysis without COSMIC genes and identified an additional 15 genomic bins containing 31 putative driver genes. These genes overlap with several OAC specific driver genes (*GATA6, MUC6*) previously identified^14^. In addition, drivers reported in other cancer types (*PVT1, THADA and YES1*) and ion channel genes (*CACNG1, CACNG4, CACNG5, KCNB1, KCNS2, KCNK6*) were identified to be preferentially affected by SVs (Supplementary Data 12).

### RUNX1 is frequently disrupted by internal deletion of exons

*RUNX1* was a candidate for a recurrent OAC driver (24% of patient samples, 92/383), uniquely affected by SVs, a known target of inter-chromosomal translocations in leukaemias, that has been shown to play a role either as an oncogene or TSG in a variety of cancer types^35–37^. It was previously reported as commonly deleted in OAC^10,38^, with a likely role as a tumour suppressor^39,40^.

**Fig. 5:**
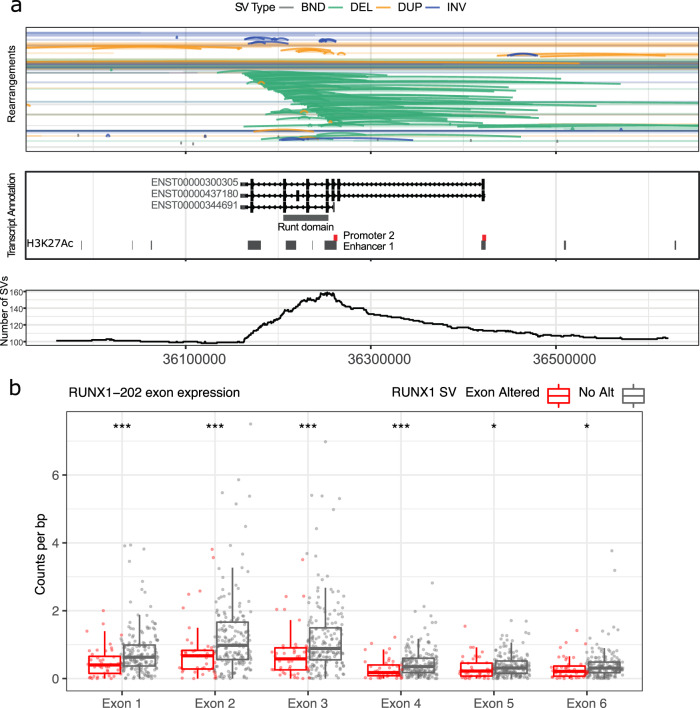
Deletions and duplications in RUNX1 affecting RUNT domain exons. **a** Genomic regions with SVs at the RUNX1 locus (arcs) with cumulative numbers of SV intervals at each position (bottom). RUNX1 is transcribed from the negative strand. RUNT domain and enhancers, from H3K27Ac data, in grey, and promoters in red. **b** Exon expression of RUNX1-202 (ENST00000344691) for with tumours with alterations in RUNX1 (red) and no alterations (grey). Read counts were normalized to length of exons. *, *** denotes *p* ≤ 0.05 and *p* ≤ 0.001 respectively. Gene structure for RUNX1-202 shown as it was determined to be highest expressed transcript by GTEx and in the cohort of 214 tumours. No transcription from promoter 1 was detected.

*RUNX1* was most commonly affected by simple SVs (60 patients) while 32 patients had complex SVs. The simple SVs comprised deletions (*n* = 53 events), duplications (*n* = 14) and inversions (*n* = 1) (Fig. 5a, Supplementary Data 11). To understand the biological effects of the RUNX1 deletions, we used data obtained from GTEX and *RUNX1* isoform expression in our cohort (Supplementary Fig. 5A) to identify the most expressed transcript (ENST00000344691) for the RUNX1 locus and showed that the most frequently deleted regions encompassed one or more of three features: an enhancer element (chr21:36250083-36262951, 65 patients), three exons (ENSE00002454902, ENSE00003519701 and ENSE00001380483, 61 patients) that code for the Runt DNA binding domain, and the promoter 2 sequence (58 patients) (Fig. 5a, Supplementary Data 13). The loss of expression of the deleted exons 1–4 were observed significantly in transcriptomic sequencing compared to unmutated tumours (Fig. 5b, Supplementary Fig. S5B). In addition, we observed that patients with promoter 2 loss have *RUNX1* expression abolished while patients with exon deletions do not show significant difference in expression compared to unmutated tumours (Supplementary Fig S5B, C).

We investigated the consequences of SVs for *RUNX1*, using PCR to confirm the genomic junctions, in 69 sequenced tumours as well as in two OAC cell lines, FLO-1 and OE33^41^. DNA was available for 17 tumours with a total of 22 RUNX1 SVs, and 20/22 (91%) were verified by PCR and Sanger sequencing, as were 3 SVs in the two cell lines (Supplementary Fig. S5D, Supplementary Data 14).

Strikingly, many of the verified SVs were predicted to preserve the reading frame of *RUNX1*, and encode a protein with absent or modified Runt domain. Most of the individual verified SV calls, 18 of 23 (including 2 of 3 SVs in cell-lines), were internal deletions or duplications that removed or duplicated exons; at least 17 of these 18 were predicted to preserve reading frame; and 15 would encode a protein with absent or modified Runt domain.

## Discussion

In this study, we identified rearrangement signatures and processes that shape the mutational and structural landscape of OAC. These encompass known DNA damage related processes including replication stress, complex rearrangements and a signature of unknown aetiology, associated with ME insertions. We estimated the contributions of ME insertions to the signature as multiple processes can result in clustered inter-chromosomal junctions. By assigning the clusters of inter-chromosomal junctions back to RS4, we found that 59% of clusters had evidence of ME insertions within the cluster. The reactivation of ME has been observed in multiple cancer types and previously been shown to associated with amplifications and deletions, most notably in *CDKN2A*^11^ and BFB events. We found that ME activity in our cohort was mainly of the Plinian type leading to a large number of retrotranspositions. Recently, expression of transposable elements has been associated to DNA damage and immune response in cancer^42^ with possible implications for targeted therapies in OAC.

Complex rearrangements were shown to be prominent in OAC in previous studies^3,9,10^ and we estimated the contributions of ME insertions and ecDNA amplicons in generating complex rearrangement clusters. The evidence of BFB cycles and ecDNA accounting for 22% of complex clusters overlapping oncogenes suggest that it is a frequent process resulting in amplifications in OACs that can undergo selection. The high copy number and expression of these amplicons, observation of enhancer hijacking and the co-amplification of multiple cancer associated genes point to a potent mechanism of tumorigenesis, often with well-known oncogenes affected^17,43–45^. Recently, mechanistic studies have shown that of telomere loss and chromosome bridge formation, generates BFB and micronuclei in in vitro systems^46^. We speculate that ecDNA can arise from multiple mechanisms in OAC including chromosome bridge formation and via the episomal model that explains the wide variety of BFB-linked and non-BFB linked ecDNA we observed in this study^18^.

In addition to SV-driven CN gains or losses, we identified the contribution of SVs to the mutational burden of known OAC drivers that would be recurrently affected by rearrangements, compared to SNVs and INDELs^14^. We adopted conventions from TCGA and ICGC to annotate SV-affecting exons in canonical transcripts of each driver gene for a conservative estimate. Notably, a substantial number of SVs encompass exons, however more work is needed to identify alterations that have strong functional effects such as a loss of protein function seen in *RUNX1*. The large overlap of candidate driver events with CN gains and losses provides a reliable way to identify patients with driver gene alterations, but poses challenges in the clinical interpretation of copy neutral variants due to inversions and translocations.

Our results suggest that, for heavily rearranged tumours, current approaches based on targeted gene panels may miss a substantial number of driver gene alterations despite inclusions of large deletions and amplifications and more work is required to identify events that are clinically relevant. For the driver genes affected by SVs, we observe that 37% are affected by gains, 33.3% losses and 4.9% have fusions involving a driver gene in either fusion partner. In addition, fusions are more likely to be associated with copy number gains (3.8%), compared to copy neutral fusions (0.4%) and losses (0.7%), The increased frequency of fusions associated with gains is likely influenced by the rearrangement process generating the SV, such as the formation of ecDNA. Overall, our findings are in keeping with the literature which suggest that fusions in OAC are rare events and few are targetable or clinically relevant.

It remains the case that there are substantial challenges for identifying and prioritizing driver genes within SVs including: (1) gene dosage effects are hard to estimate as complex CN changes such as whole genome doubling are present in the majority of tumours; (2) complex SVs affecting driver genes can encompass large regions with multiple passenger genes implicated; and (3) downstream effects of SV events are hard to determine and need to be validated experimentally. In our analysis we focused on driver genes in OAC and other cancers, as these genes often coincide with the recurrence of SVs within a large genomic region. We used additional evidence such as the patterns of SVs, focal deletions and duplications spanning the gene to identify the driver gene affected by SVs.

We have further identified regions in the genome with a high recurrence or density of SVs that were likely to undergo selection. The analysis recapitulated driver genes identified previously in OAC and pan-cancer studies^1,4,14^. Several drivers including *AKAP9, CDK12, RARA, CCR7* were associated in co-amplification of regions that were part of BFB and ecDNA while *MYB* was identified in breast and OAC to be affected by amplification in coding regions. *BCL3* has been previously identified as a transcriptional activator in leukaemias and has recently been shown to activate an array of pathways including WNT and NFKB^47^. *RUNX1* was mainly affected by CN loss and rearrangements.

Our analysis of *RUNX1* rearrangements suggests that the most frequent events in OAC either result in promoter loss or remove or duplicate internal exons, so that a RUNX1 protein would still be encoded but with the RUNT domain disabled—the domain that mediates DNA binding and heterodimerisation with other transcription factors^35^. Although *RUNX1* mutations and rearrangements have been described in OAC^39,48,49^ and other carcinomas^35,50,51^ to our knowledge this consequence of mutation has not been noted before, except in a single example of an in-frame deletion of genomic exon 6 in the breast cancer cell line HCC1937^50^. Our data suggest that mutations of *RUNX1* in OAC, and perhaps in other carcinomas, are change-of-function, rather than simple gain or loss-of-function. There are, however, tumours that appear to have simply lost *RUNX1* activity, so *RUNX1* may be altered in a variety of ways, reflecting RUNX1’s role as a master regulator of cell fate, with ability to interact with various other transcription factors and chromatin modifiers^35,36^.

Our analysis provide evidence for the processes leading to heavily rearranged genomes which are a hallmark of OAC. In addition, these rearrangements confer varying degrees of selective advantage and different evolutionary trajectories. By understanding the mechanisms underlying the formation of SVs, it is hoped that in the future we can identify patients that have a better prognosis and develop therapy regimes that exploit the same tumorigenic processes.

## Methods

### Study design, cohort selection and sequencing

Endoscopic biopsies and resection specimens were collected prospectively from 383 oesophageal adenocarcinoma patients, including 83 cases previously included in the ICGC pan-cancer (PCAWG) studies^3^. Patients were predominantly male (*n* = 329, 86%), with a median age at diagnosis of 66.8 years (IQR: 59–73.6), and presented at an advanced stage (T3N2 = 56.15%, T3N1 = 47.12%). All cases had an estimated tumour purity of >70%, following expert pathological review and underwent whole genome sequencing by Illumina using 100-150 bp paired end reads with 50-fold coverage for the tumour and 30-fold coverage for the matched germline control. Reads were mapped to the GRCh37/hg19 reference assembly using BWA-mem^52^ (v0.7.17). Paired end RNA-Seq with 75-bp read length was performed for a subset of 214 tumours that had sufficient material^14^. The RNA-Seq data were aligned using STAR^53^ (v2.6.1d) and reads mapped to each gene was counted using the GenomicAlignments^54^ (v1.20.1) R package using Ensembl release 87 annotation. Transcript per million values (TPM) were calculated and used in logistic regression modelling. Transcript isoform and exon expression quantification were carried using Kallisto^55^ (0.46.1) and subread^56^ (v 2.0.3) and normalized using the lengths of transcript and exon, respectively.

### Structural variation calling and validation

SVs were called, after alignment with bwa-mem to GRCh37/hg19 (1000 Genomes Project human_g1k_v37 with decoy sequences hs37d5), using MANTA v0.27^57^, as junctions that resembled deletions (DEL), inter-chromosomal junctions (BND), duplications (DUP), or inversions (INV). We discarded SVs that had any supporting reads in the matched normal; SVs found in a pool of 50 unmatched normals from peripheral blood and 15 samples from distant oesophageal mucosa.

The filtered Manta SV calls were compared to calls made by the ICGC Pan cancer project^1,3^ which used four other pipelines: dRanger and Snowman (Broad Institute), DELLY (DKFZ), and BRASS (Wellcome Sanger Institute), for the 100 of our oesophageal adenocarcinomas included in the PCAWG project. We identified equivalent calls with mergevcf allowing a 300 base pair difference in coordinates, and each individual pipeline was compared to a consensus sets which included variants called by at least two of the ICGC pipelines. Our Manta pipeline gave a median precision of 0.92, a median sensitivity of 0.89, and a median similarity of 0.82. Alternative sets of equivalent calls were also identified allowing base pair differences of 100 and 500; the *F*1 score for our Manta pipeline was the highest of all of the five pipelines at all three base pair windows sizes.

We also selected a representative tumour sample and PCR verified 73/91 (80%) randomly selected SVs identified by our pipeline (Supplementary Data 15). Additionally, we verified the breakpoints in the coding sequence of two recurrently rearranged genes and confirmed the rearrangement in 79% (15/19) and 74% (20/27) of the cases respectively (Supplementary Data 15). For an overview of the analyses and software, see Supplementary Fig. S6.

### Mobile element calling

To identify mobile element (ME) insertions independently of SV calling we used TraFiC-mem v1.1.0 (https://gitlab.com/mobilegenomes/TraFiC)^11,13^ with the MEIBA (https://github.com/brguez/MEIBA/tree/master/src/python) module to give base-pair resolution, and discarding inserts that lacked the expected poly-A tail. These inserts were used to annotate BNDs as ME insertions if either breakend directly overlapped with insert regions. In addition, breakpoints in sequences known to be transduced by LINE-1 mobile elements^12,13^ were marked as likely mobile element insertions.

### Classification of SV footprints and rearrangement signature analysis

We classified SVs into footprints by identifying clusters as described^1^ using the ClusterSV R package (https://github.com/cancerit/ClusterSV). In addition, clusters of ME transductions were defined as clusters containing BNDs with at one breakpoint overlapping with ME insertions. We then set aside with ME footprints and classified the remaining SVs as simple or complex rearrangements after excluding centromere and telomere regions.

Rearrangement signatures (RS) were extracted using the Palimpsest 1.0.0 R package^23^, Palimpsest was run for 1000 iterations for from 2 to 10 signatures, and six signatures were selected based on cophenetic and silhouette scores. We matched the extracted signatures to reference rearrangement signatures^24^ from Signal (https://signal.mutationalsignatures.com/, Supplementary Fig S1A, B). We clustered patients based on the exposures of the extracted SV signatures using the ConsensusClusterPlus^58^ (v1.46.0) R package. The final number of clusters (*K*) was chosen using the calcICI function with the *K* = 6 selected, based on the highest mean consensus score.

### Chromothripsis, extrachromosomal amplicons and break–fusion-bridge events

Chromothripsis was identified as complex SV events with oscillating copy number changes, using ShatterSeek v0.4^21^, and classified as high confidence (≥7 segments with oscillating copy number) or low confidence (4–6 segments) as recommended.

Regions resembling extrachromosomal amplifications or breakage–fusion-bridge cycles were identified using AmpliconArchitect v1.2^15^: amplifications of size 50 kb, copy number > 4.5 were reconstructed using CNVKit^29^ v0.9.8 called copy number segments. Amplified segments were refined with the *amplified_intervals.py* script. AmpliconArchitect was run using the *clustered* mode to identify extrachromosomal regions with driver gene amplifications and fold-back events associated with breakage–fusion-bridge cycles.

### Rearrangement signature features and regression

To identify features associated to each RS, we carried out logistic regression using the glm function in R (stats R package) based on the presence of each RS as response and predictors including: number of ME insertions, chromothripsis events, complex SV clusters, mutations attributed to SNV signatures extracted using SigProfilerExtractor v1.1.0^59^ listed by the COSMIC database^60^, mutational signature subtypes^10^, total BFB or ecDNA events and gene expression of known driver genes (Supplementary Data 3).

We log transformed and scaled the counts from the predictors. For each RS, predictors from the univariate analysis with *p* < 0.05 were used to build a multivariate model, refined with stepAIC (MASS R package, version 7.3-51.1) and FDR correction was done on the final model. In addition, we carried out a hold-out validation of 10 replicates each using 80–20, 60–40 and 40–60 split of the cases with each signature and observed that positive associations between RS4-ME events, RS9-DDR subtype, RS6 + 12-BFB, RS7−SBS17a and negative associations between RS1-ec_CDK6, RS7-ME events were robust throughout each hold-out validation (Supplementary Data 16). A separate logistic regression model was built using the RNA-Seq gene expression profiles in SV driver genes (Supplementary Data 4, 11).

In addition, we carried out a correlation matrix analysis on the response and predictors using the rcorr function (Hmisc R package, version 4.2-0) and carried out FDR correction on the final *p*-values. All associations except for RS9-DDR, RS9-Mutagenic, RS9-CCNE1 and RS2-KIF5B were validated using the correlation matrix analysis (Supplementary Data 4).

### Estimating the contributions of SV in known drivers

To estimate the contributions of SV in recurrent drivers, we defined regions between two SV breakpoint called by MANTA and identified SVs with regions that overlapped exons in known driver genes from Frankell 2019 and Campbell 2020. To identify gene isoforms that are likely affected, we used annotations (vcf2maf tool, isoform_overrides_uniprot) from TCGA to select for overlaps in exons present in canonical transcripts of each gene. The predominant isoforms for RUNX1 were obtained from the GTEx database using the oesophagus mucosa and stomach tissue types.

In addition, GISTIC 2.0^61^ was used to identify gains, amplifications, loss or deep deletions in genes in addition to SNVs, INDELS and SV.

### Identifying regions of frequent SVs

We divided the genome into 1 Mb bins with 500 kb overlap and calculated breakpoint *recurrence*, i.e. the number of patients with at least one breakpoint in the bin, and breakpoint *density*, the average number of breakpoints in each bin across all samples (Fig. 3a).

To estimate the background SV rate^19,32^, we modelled breakpoint recurrence in each bin as a negative binomial linear regression, adjusted for the genomic context of each bin: fragile sites, copy number aberrations, GC content, replication timing^62^, histone methylation marks (H3K36me3 and H3K27ac), DNAseq hypersensitivity, and ALU sequences^19^. Bins were identified as being significantly recurrently altered if the residuals were ≥2 standard deviations from the mean (Supplementary Data 10).

In order to further characterize bins that may reflect hotspots for SV activity we filtered bins that reflect known fragile sites and high-density regions (434/5597 bins). We then apply three methods to identify focal hotspots and select bins found by at least two methods: (1) model the per-bin SV counts genome-wide under a negative binomial distribution identifying the residual outliers as significant bins, (2) model the per-bin SV counts per-chromosome to account for chromosomal context, and (3) a rank-sum approach where counts are ranked per-patient and summed across each bin and significant bins identified via *k*-means clustering.

To identify driver genes enriched in tumours with RS4 compared to tumours devoid of MEs (RS7 enriched tumours), we calculated the frequency of tumours with SVs in 1 Mb bins in RS4 and RS7 tumours (93 and 76, respectively) and identified those with a frequency difference of ≥15% between groups. We excluded fragile sites for this analysis.

### Statistics and reproducibility

Statistical tests were carried out using R 3.5.3, with the wilcox.test function for the Wilcoxon rank sum test to associate biological features and each RS (*n* = RS9:261, RS1:180, RS7:266, RS4:283, RS2:335, RS6a + 12:264) and between RS patient groups (*n* = RS2:119, RS7 + 9:106, RS6a + 12:74, RS4:42, RS1:42). All reported Wilcoxon rank sum tests *p*-values are two tailed.

Permutation tests for the enrichment of H3K27AC enhancer elements in ecDNA regions were carried out using the regioneR^30^ package and overlapPermTest function with 5000 permutations. Two proportions *z*-test to compare recurrence of driver genes (*n* = 383) was carried out using the prop.test function with the alternative = ‘greater’ parameter, followed by multiple testing correction using p.adjust(method = ‘fdr’).

### Reporting summary

Further information on experimental design is available in the Nature Research Reporting Summary linked to this paper.

## Supplementary information

Supplementary Figures

Description of Additional Supplementary Files

Supplementary Data 1

Supplementary Data 2

Supplementary Data 3

Supplementary Data 4

Supplementary Data 5

Supplementary Data 6

Supplementary Data 7

Supplementary Data 8

Supplementary Data 9

Supplementary Data 10

Supplementary Data 11

Supplementary Data 12

Supplementary Data 13

Supplementary Data 14

Supplementary Data 15

Supplementary Data 16

Reporting summary

## Data Availability

The sequencing data included in this study have been submitted to European Genome-phenome Archive (EGA; https://ega-archive.org/) under the accession numbers EGAD00001007808 (WGS) and EGAD00001007809 (RNAseq), respectively.

## Code availability

R scripts used in the analyses are available on GitHub (https://github.com/fitzgerald-lab/Rearrangements-in-OAC).
