# Supplementary material for: Rearrangement processes and structural variations show evidence of selection in oesophageal adenocarcinomas
Source: Commun Biol. 2022 Apr 8;5:335. doi: 10.1038/s42003-022-03238-7 (PMC8993906; doi:10.1038/s42003-022-03238-7)

Supplementary Figures

	Supplementary Figures	Page
S1	SV signatures extracted from 383 OAC tumours and comparison to Signal reference rearrangement signatures	2
S2	SV signature group correlations	3
S3	Enhancer regions amplified in ecDNA amplicons	4
S4	Distribution of SV hotspots containing driver genes	5
S5	RUNX1 isoform expression analysis and PCR validations	6
S6	Overview of analyses and software	7

Supplementary_Fig_S1 A) SV Signatures extracted from 383 OAC and
B) Comparison with Signal reference rearrangement signatures

A

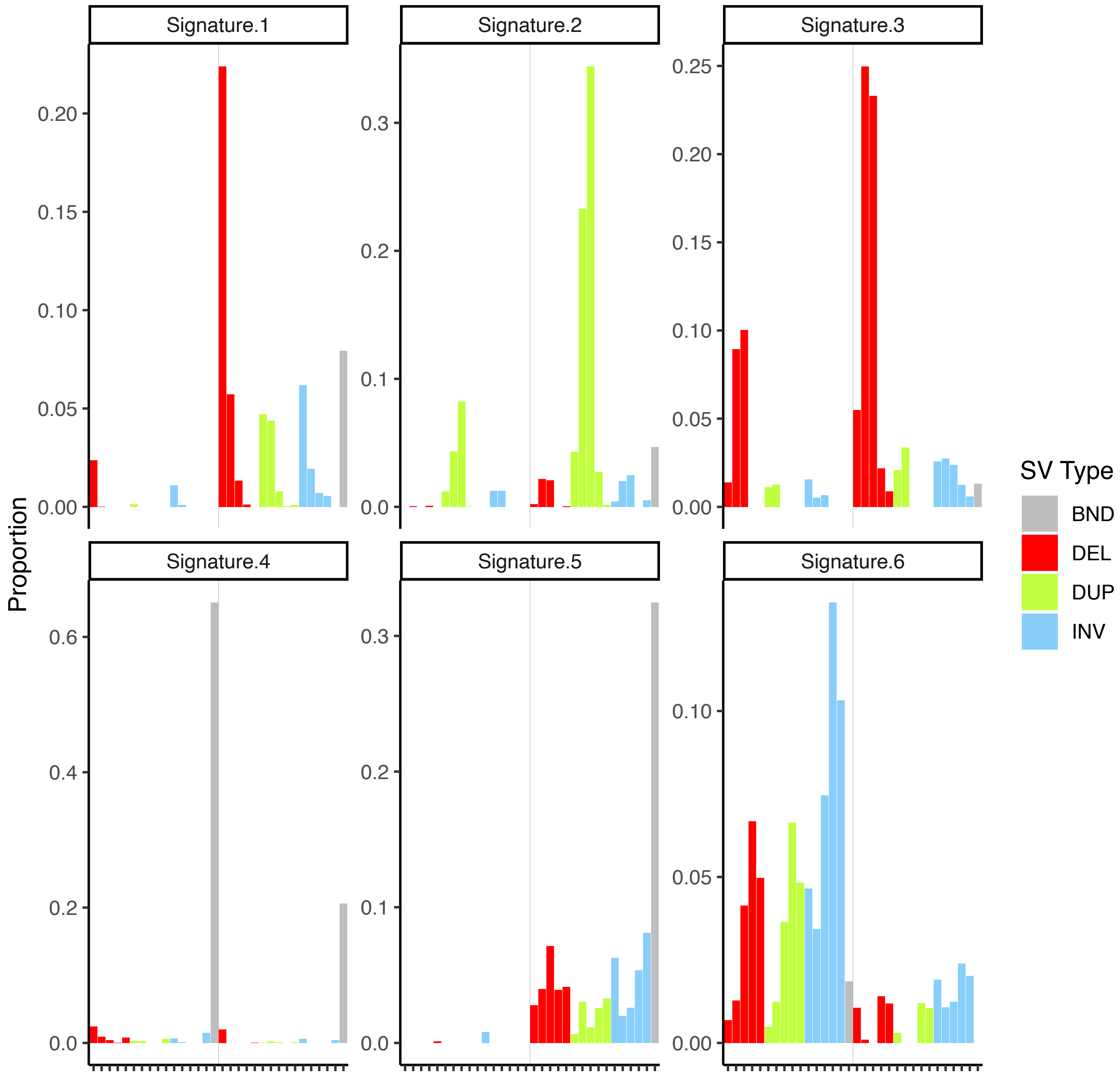

B

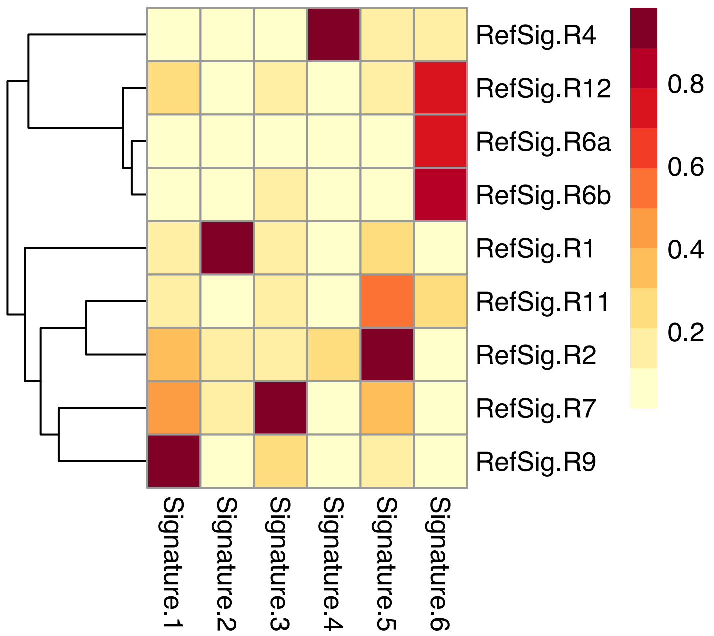

Supplementary_Fig_S2. SV signature group correlations

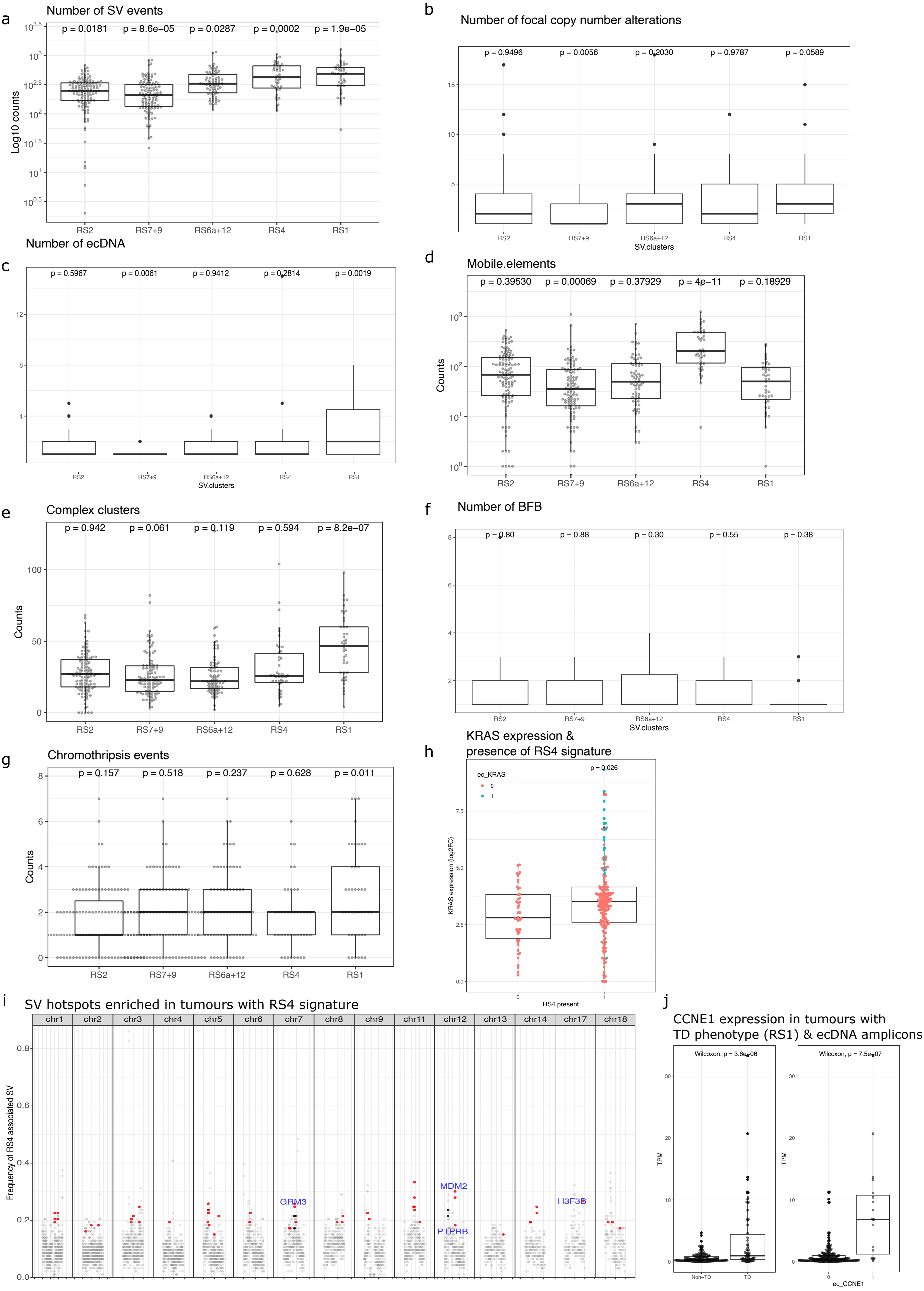

Supplementary_Fig_S3. Enhancer regions amplified in ecDNA amplicons. Four example of amplified regions involved in ecDNA formation (top), expression of genes from the same sample (middle) with driver genes highlighted (red) and H3K27Ac peaks identified in tumours and tumour cell lines from Chen et al (2019).

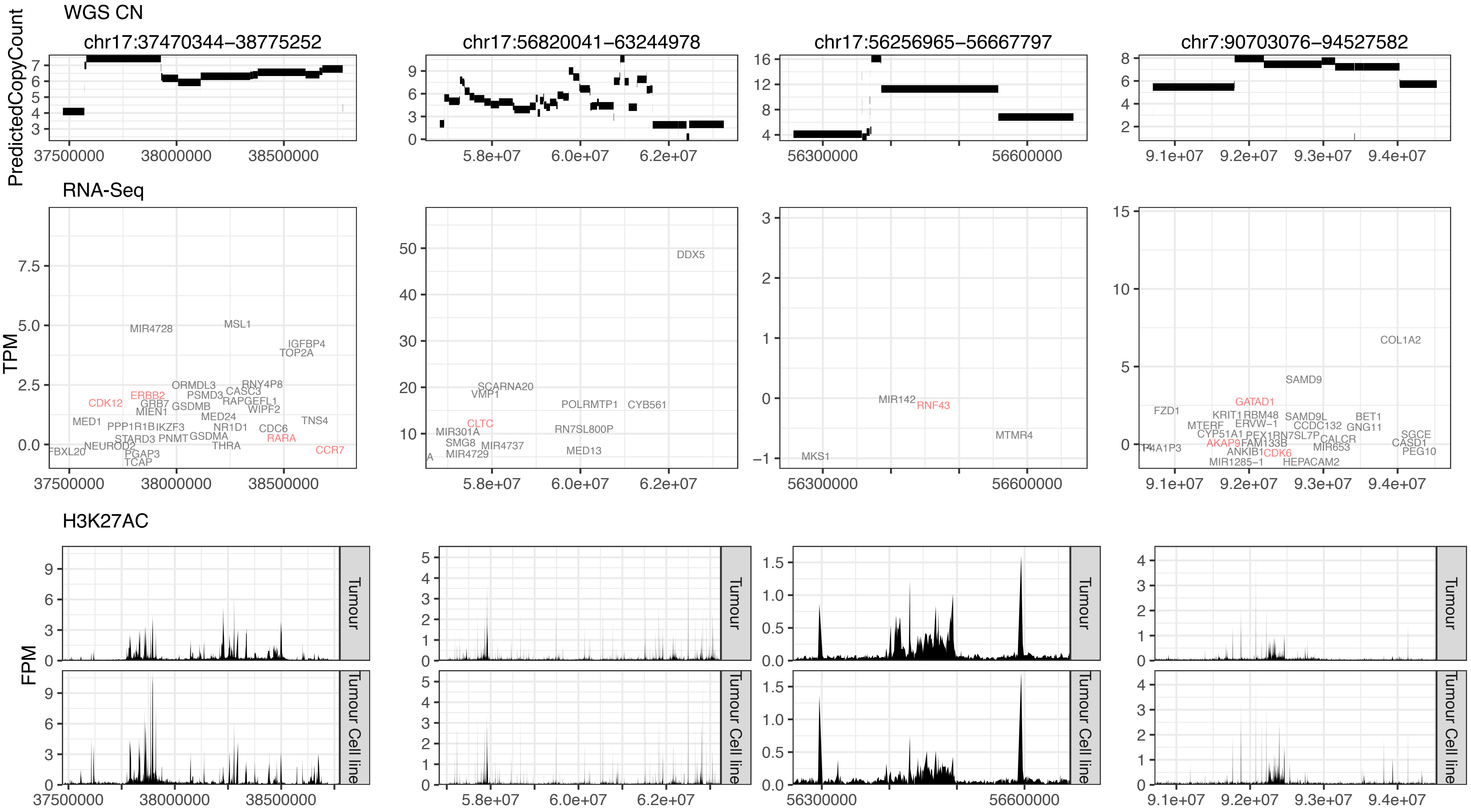

Supplementary_Fig_S4 Distribution of SV in hotspot containing BCL3. SVs in A) driver hotspot bin shown as arcs (within bin) and lines (spanning bin). Positions of enhancers and genes showed with driver gene region highlighed (red).

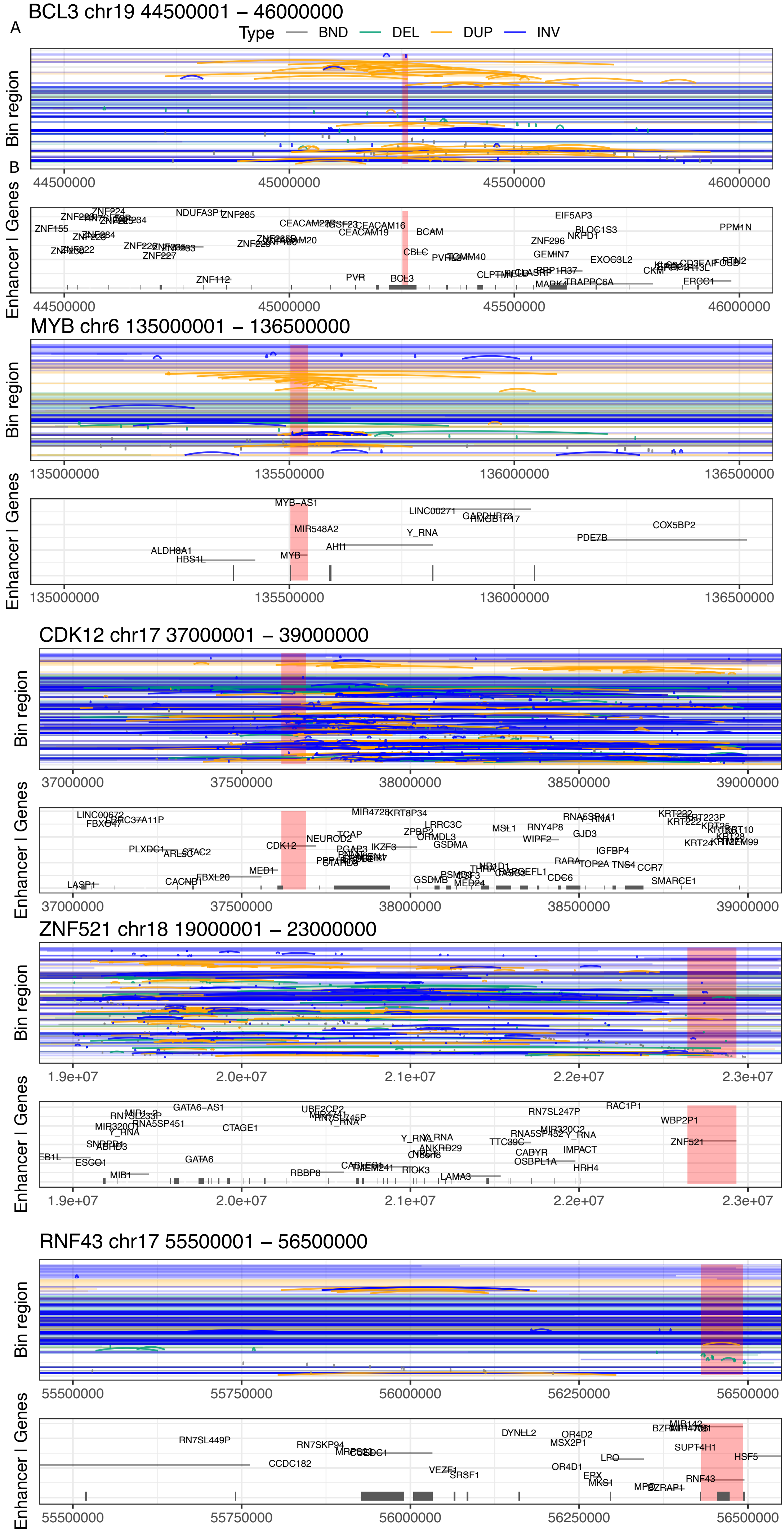

Supplemental_Fig_S5.RUNX1 isoform expression and PCR validation

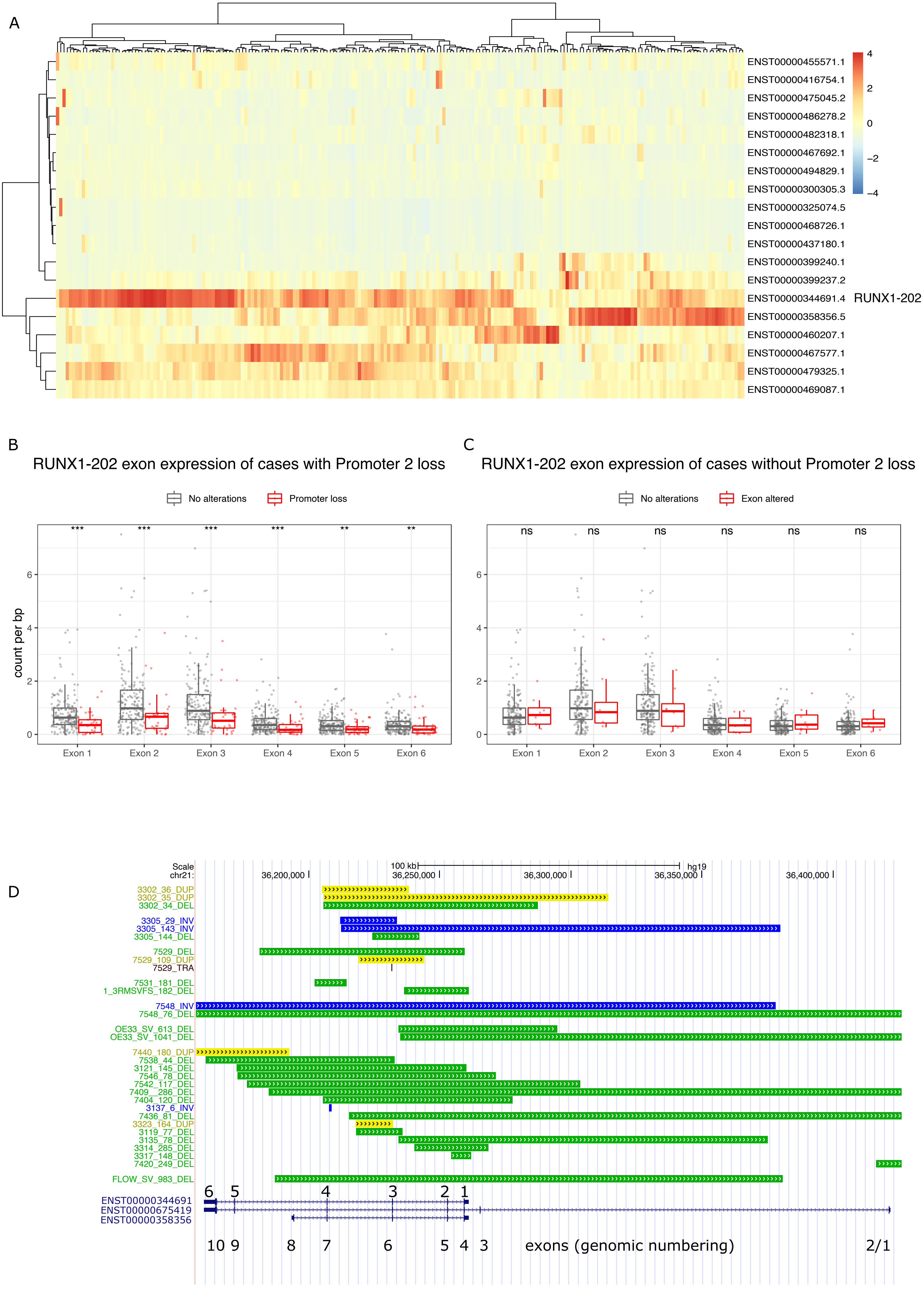

Supplemental_Fig_S6. Overview of analyses and software

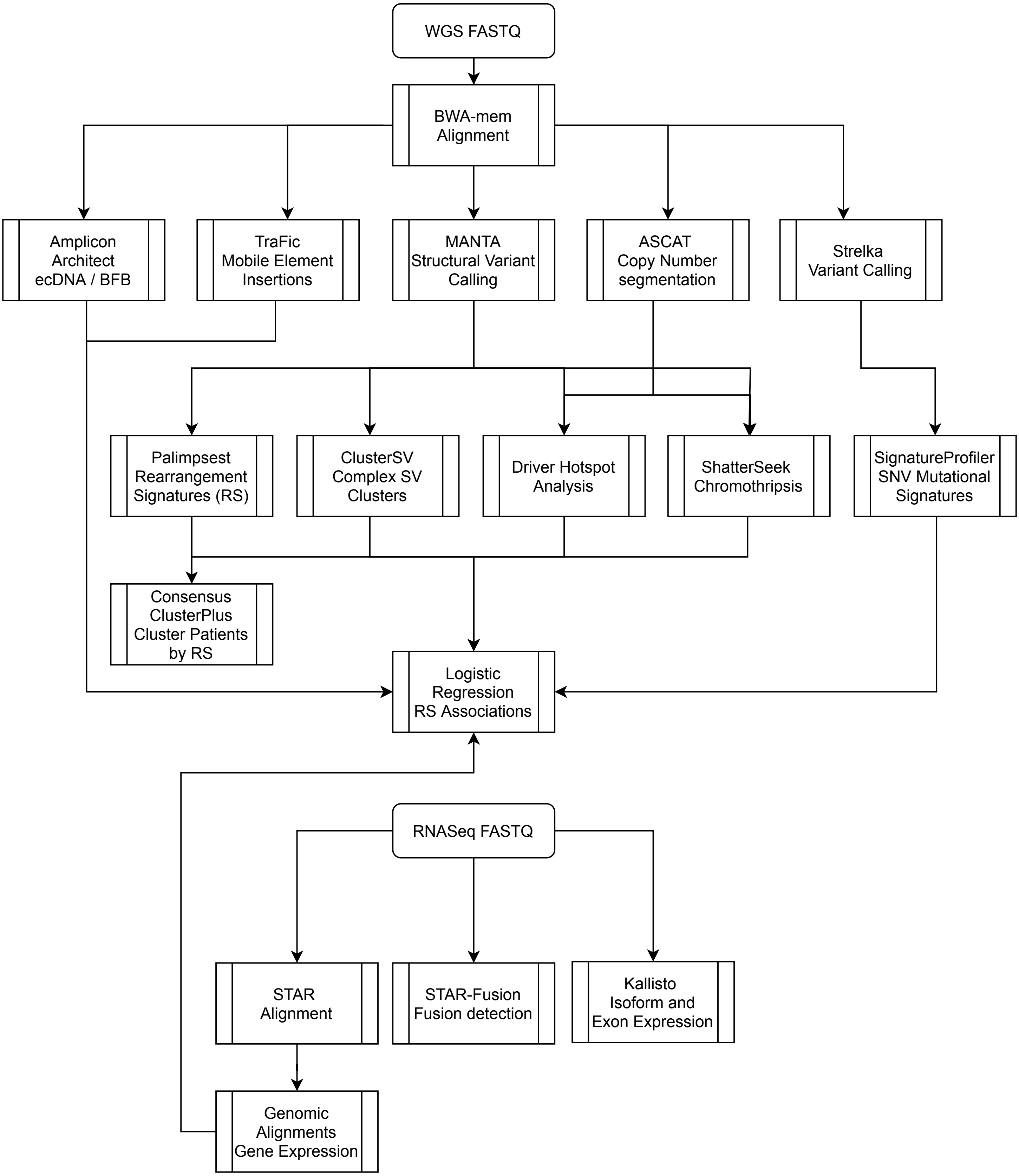

Supplement: Supplementary file 1 — Supplementary Figures [file 42003_2022_3238_MOESM1_ESM.pdf]
