# Supplementary material for: Rearrangement processes and structural variations show evidence of selection in oesophageal adenocarcinomas
Source: Commun Biol. 2022 Apr 8;5:335. doi: 10.1038/s42003-022-03238-7 (PMC8993906; doi:10.1038/s42003-022-03238-7)
Supplement: Supplementary file 2 — Description of Additional Supplementary Files [file 42003_2022_3238_MOESM2_ESM.pdf]

Description of additional supplementary files

File name: Supplementary Data 1
Description: Summary of SV signature burden and correlates

File name: Supplementary Data 2
Description: Summary of ME insertions identified by TraFic

File name: Supplementary Data 3
Description: Variables used for logistic regression analysis

File name: Supplementary Data 4
Description: Output of logistic regression of SV signatures

File name: Supplementary Data 5
Description: Summary of SV clusters, ME and Amplicon Architect intervals

File name: Supplementary Data 6
Description: List of tumours with KRAS ecDNA and number of ME clusters

File name: Supplementary Data 7
Description: Enhancer regions with no annotated genes in ecDNA amplicons

File name: Supplementary Data 8
Description: Amplicon copy number and gene expression

File name: Supplementary Data 9
Description: Two proportions z test for driver genes altered by SVs

File name: Supplementary Data 10
Description: SV driver hotspots

File name: Supplementary Data 11
Description: Breakdown of cases and SV types in SV driver genes

File name: Supplementary Data 12
Description: Non-COSMIC driver SV

File name: Supplementary Data 13
Description: RUNX1 exons and enhancers deleted in 79 patients

File name: Supplementary Data 14
Description: PCR validated SVs in RUNX1 in tumours and cell lines

File name: Supplementary Data 15
Description: PCR validation of SV events

File name: Supplementary Data 16
Description: RS Regression covariates with hold out
