# Supplementary material for: Rearrangement processes and structural variations show evidence of selection in oesophageal adenocarcinomas
Source: Commun Biol. 2022 Apr 8;5:335. doi: 10.1038/s42003-022-03238-7 (PMC8993906; doi:10.1038/s42003-022-03238-7)
Supplement: Supplementary file 19 — Reporting summary [file 42003_2022_3238_MOESM19_ESM.pdf]

nature research

Corresponding author(s): Rebecca C. Fitzgerald

Last updated by author(s): 18/02/2022

Reporting Summary

Nature Research wishes to improve the reproducibility of the work that we publish. This form provides structure for consistency and transparency in reporting. For further information on Nature Research policies, see our Editorial Policies and the Editorial Policy Checklist.

Statistics

For all statistical analyses, confirm that the following items are present in the figure legend, table legend, main text, or Methods section.

n/a	Confirmed	
☐	☒	The exact sample size (*n*) for each experimental group/condition, given as a discrete number and unit of measurement
☐	☒	A statement on whether measurements were taken from distinct samples or whether the same sample was measured repeatedly
☐	☒	The statistical test(s) used AND whether they are one- or two-sided<br>*Only common tests should be described solely by name; describe more complex techniques in the Methods section.*
☐	☒	A description of all covariates tested
☐	☒	A description of any assumptions or corrections, such as tests of normality and adjustment for multiple comparisons
☐	☒	A full description of the statistical parameters including central tendency (e.g. means) or other basic estimates (e.g. regression coefficient) AND variation (e.g. standard deviation) or associated estimates of uncertainty (e.g. confidence intervals)
☐	☒	For null hypothesis testing, the test statistic (e.g. *F*, *t*, *r*) with confidence intervals, effect sizes, degrees of freedom and *P* value noted<br>*Give P values as exact values whenever suitable.*
☒	☐	For Bayesian analysis, information on the choice of priors and Markov chain Monte Carlo settings
☐	☒	For hierarchical and complex designs, identification of the appropriate level for tests and full reporting of outcomes
☐	☒	Estimates of effect sizes (e.g. Cohen's *d*, Pearson's *r*), indicating how they were calculated

Our web collection on statistics for biologists contains articles on many of the points above.

Software and code

Policy information about availability of computer code

Data collection	No software was used
Data analysis	BWA-mem v0.7.17 , STAR v2.6.1d, GenomicAlignments v1.20.1, Kallisto 0.46.1, subread v2.0.3 , MANTA v0.27, TraFiC-mem v1.1.0 , ClusterSV, Palimpsest v1.0.0, ConsensusClusterPlus v1.46.0, ShatterSeek v0.4, AmpliconArchitect v1.2, CNVKit29 v0.9.8, SigProfilerExtractor v1.1.0, MASS R package, v7.3-51.1, GISTIC 2.0, Hmisc R package v4.2-0

For manuscripts utilizing custom algorithms or software that are central to the research but not yet described in published literature, software must be made available to editors and reviewers. We strongly encourage code deposition in a community repository (e.g. GitHub). See the Nature Research guidelines for submitting code & software for further information.

Data

Policy information about availability of data

All manuscripts must include a data availability statement. This statement should provide the following information, where applicable:

- Accession codes, unique identifiers, or web links for publicly available datasets
- A list of figures that have associated raw data
- A description of any restrictions on data availability

The sequencing data included in this study have been submitted to European Genome-phenome Archive (EGA; https://ega-archive.org/) under the accession numbers EGAD00001007808 (WGS) and EGAD00001007809 (RNAseq) respectively.

Field-specific reporting

Please select the one below that is the best fit for your research. If you are not sure, read the appropriate sections before making your selection.

☒ Life sciences ☐ Behavioural & social sciences ☐ Ecological, evolutionary & environmental sciences

For a reference copy of the document with all sections, see nature.com/documents/nr-reporting-summary-flat.pdf

Life sciences study design

All studies must disclose on these points even when the disclosure is negative.

Sample size	No sample size was chosen and maximum number of available samples were used. A sample size of 383 tumours with whole-genome sequencing data was selected, with a subset of 214 tumours with RNA sequencing data available from the same specimen
Data exclusions	No data exclusions
Replication	PCR validation of randomly selected structural rearrangement events and specifically for the RUNX1 was done.
Randomization	Not relevant
Blinding	Not relevant

Reporting for specific materials, systems and methods

We require information from authors about some types of materials, experimental systems and methods used in many studies. Here, indicate whether each material, system or method listed is relevant to your study. If you are not sure if a list item applies to your research, read the appropriate section before selecting a response.

Materials & experimental systems

n/a	Involved in the study	
☒	☐	Antibodies
☒	☐	Eukaryotic cell lines
☒	☐	Palaeontology and archaeology
☒	☐	Animals and other organisms
☐	☒	Human research participants
☒	☐	Clinical data
☒	☐	Dual use research of concern

Methods

n/a	Involved in the study	
☒	☐	ChIP-seq
☒	☐	Flow cytometry
☒	☐	MRI-based neuroimaging

Human research participants

Policy information about studies involving human research participants

Population characteristics	Endoscopic biopsies and resection specimens were collected prospectively from 383 oesophageal adenocarcinoma patients. Patients were predominantly male (n=329, 86%), with a median age at diagnosis of 66.8 years (IQR: 59-73.6), and presented at an advanced stage (T3N2 = 56,15%, T3N1 = 47,12%).
Recruitment	Patients are recruited after diagnosis of oesophago-gastric cancer and samples taken at times of clinically indicated interventions either at the time of surgery or before using biopsies.
Ethics oversight	The study was registered (UKCRNID 8880), approved by the Institutional Ethics Committees (REC 07/H0305/52 and 10/H0305/1), and all participants gave written informed consent as part of the OCCAMS (Oesophageal Clinical And Molecular Stratification) consortium.

Note that full information on the approval of the study protocol must also be provided in the manuscript.
